# Artificial intelligence explainability: the technical and ethical dimensions

**DOI:** 10.1098/rsta.2020.0363

**Published:** 2021-10-04

**Authors:** John A. McDermid, Yan Jia, Zoe Porter, Ibrahim Habli

**Affiliations:** Department of Computer Science, University of York, Deramore Lane, York YO10 5GH, UK

**Keywords:** explainability, machine learning, assurance

## Abstract

In recent years, several new technical methods have been developed to make AI-models more transparent and interpretable. These techniques are often referred to collectively as ‘AI explainability’ or ‘XAI’ methods. This paper presents an overview of XAI methods, and links them to stakeholder purposes for seeking an explanation. Because the underlying stakeholder purposes are broadly ethical in nature, we see this analysis as a contribution towards bringing together the technical and ethical dimensions of XAI. We emphasize that use of XAI methods must be linked to explanations of human decisions made during the development life cycle. Situated within that wider accountability framework, our analysis may offer a helpful starting point for designers, safety engineers, service providers and regulators who need to make practical judgements about which XAI methods to employ or to require.

This article is part of the theme issue ‘Towards symbiotic autonomous systems’.

## 1. Introduction

Increasingly, artificial intelligence (AI)—specifically, machine learning (ML)—is being used in ‘critical’ systems. Critical systems directly affect human well-being, life or liberty. These may be digital systems (such as those that are used by human experts to inform decisions regarding medical treatment or prison sentences) or embodied autonomous systems (such as highly automated cars or unmanned aerial vehicles). The use of critical ML-based systems to assist or to replace the human decision-maker raises many questions about when, and whether, we should trust them. AI explainability (XAI) methods are part of the answer. The use of XAI methods can contribute to building assurance, or justified confidence, in critical systems employing ML.

In this paper, we will link stakeholder purposes and the technical dimensions of explainability. Stakeholders may seek to use XAI methods for a range of reasons, such as to assess confidence, to inform consent, to contest decisions or to regulate the use of systems. These reasons are often broadly ethical in nature. We argue that XAI methods are one way to help serve these purposes, but the requirement for justification also traces the explananda back to human decisions during design and implementation. Thus, XAI methods sit within a wider accountability ecosystem. Our approach has similarities to [1] in focusing on the stakeholder groups who comprise the audience for XAI methods, but our focus is more distinctly on the practical reasons for which stakeholders seek an explanation; we also add some stakeholder classes, such as prediction-recipients, as distinguished from end-users and the courts. Our approach also has similarities with [2] in that it identifies variations in requirements for explanations across stakeholders, but we focus more on *external* stakeholders, e.g. regulators, given our greater emphasis on safety and assurance.

The remainder of the paper is structured as follows. Section 2. introduces and contextualizes the explainability of ML-based systems. Section 3. identifies key stakeholder classes, and it considers the time dimension and general underlying purposes stakeholders will likely have for XAI methods. This helps to structure an analysis of the state of the art in explainabilty; §4. surveys widely used global and local XAI methods, categorizing the latter as either feature-importance or example-based methods. Section 5. illustrates some of these methods in use for a clinical Decision-Support System (DSS). The integration of the analysis of stakeholder purposes and XAI methods then occurs in §6. which includes a table that cross-references the needs of stakeholders against the XAI methods available. This is supported by a narrative description of three scenarios to deepen understanding. Section 7. takes a systems engineering perspective, discussing trade-offs between explainability and performance, and addressing the broader role of XAI methods in safety assurance. Section 8. considers the importance of explainability in achieving and assuring trustworthy AI and ML.

## 2. Explaining explainability

### (a) The challenge of AI explainability

Conceptually, traditional software development follows a defined ‘life cycle’. It starts with the definition of requirements, proceeds via design to implementation, e.g. coding, and then the software is progressively tested as individual parts of the software are integrated to make up the overall system. Where systems are critical, the life cycle is very rigorous. Key requirements, e.g. for safety, are defined and refined at each stage of the development. Verification gives *assurance* that the system meets its key requirements; McDermid [3] illustrates this process for safety-critical software in aviation. Typically, where there is a formal regulatory system, standards define what needs to be done to achieve assurance and to gain approval for deployment of the system.

By contrast, development of ML-based systems is a highly *iterative* process, with a very different life cycle and the current standards do not give a basis for assurance. The models at the heart of ML-based systems are trained on data representative of the problem to be addressed and then their performance is evaluated against pre-defined criteria, e.g. the number of false positives in detecting tumorous growths in X-ray images, and refined until their performance is satisfactory. The models have utility because they generalize beyond their initial training data. For example, autonomous vehicles (AVs) can identify pedestrians in situations that were not present in their training dataset, predict their trajectory and carry out manoeuvres to avoid a collision.

There are many classes of ML, e.g. neural networks (NNs) [4], support vector machines (SVMs) [5] and (deep) reinforcement learning (RL) [6]. NNs have sub-classes, e.g. convolutional neural networks (CNNs) [7] and deep neural networks (DNN) [8]. Our aim here is to ‘explain explainability’ so far as practicable without going into details of particular ML methods. For our purpose here, we can characterize ML-based systems as being trained on large datasets to perform classification or regression tasks. When the resulting models are used for classification purposes, they make probabilistic predictions, e.g. a 90% probability that an image contains a tumour.

ML models are often highly complex and thus are not directly amenable to human inspection (alternatively ‘opaque’ or ‘black boxes’). Further, the ML model structure may not match the features humans would use in making the decisions, so interpretation would remain difficult even if the model could be inspected. Some image analysis systems can make erroneous classifications of objects when a small amount of noise is added which is imperceptible to a human—but very significant in the model because of the features that have been learnt [9].

In simple terms, XAI methods seek to provide human interpretable representations of the ML models to help overcome these and other problems.

### (b) Context and roles for explainability

**Figure 1.**
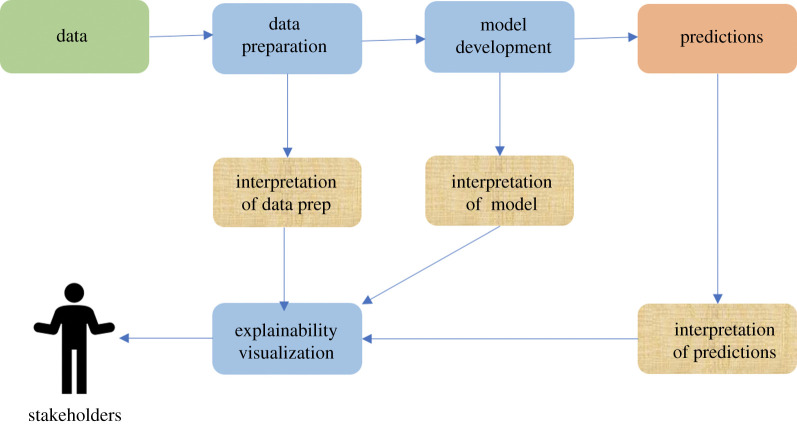
Context and roles of explainability. (Online version in colour.)

We use a simple illustration, based on the ML life cycle model in [10], to ‘explain explainability’ (figure 1). This figure is intended to show that different stakeholders, e.g. users, regulators and courts, may have different *purposes* in trying to understand what the ML-based system is doing. The intent is that the information is presented to the stakeholder in a meaningful context. This, in turn, can help to inform human decision-making, e.g. deciding whether or not to approve the use of an ML-based system or to accept a prediction or recommendation.

The boxes shaded in gold-brown in figure 1 indicate what explanations may be needed. XAI research in the technical community mainly focuses on explaining the system’s outputs (predictions) and on the model. But there will also often be a need to explain the collection of the data (box shaded in green), and preparation of the training data to show that it is balanced, e.g. in terms of gender or race, or to show that it covers *all* the different sorts of road junction found in a given country where an AV is to be used. Data preparation is a key explanandum of an ML model, and the human decision makers should be able to explain the choice of the particular dataset, which is the first step in the ML life cycle.

ML models reflect features of the problem which the system is intended to solve (and of its solution). In practice, the ML system developer shapes the set of features represented in the model by training it on selected data, assessing its performance (e.g. what proportion of pedestrians it correctly recognizes—known as true positives—and those objects, such as life-size pictures of people in an advert on the side of a bus, it mistakenly categorizes as pedestrians—known as false positives), and iterating to improve performance. Training will seek to balance the performance between the different criteria. This balance will be decided by the developer for a particular system, e.g. for an AV, a high level of false positives may be acceptable to reduce false negatives, for safety reasons. Development of ML models always involves such balances or trade-offs. What XAI methods can do is to highlight the consequences of such trade-offs. In fact, a lot of work on explainability, e.g. [2], is focused on developers to help them guide ML-model development but, in this paper, we will primarily focus on other stakeholders, external to the development.

To avoid confusion with the decisions made by humans in-the-loop, the term ‘predictions’ is used in figure 1 but these might include decisions made by an autonomous system, e.g. an AV may decide to stop when it detects a traffic light at red. We continue with this terminology throughout the rest of paper: all outputs of the ML-based system—whether decisions, recommendations, predictions or classifications—will be referred to as ‘predictions’.

### (c) Types of explainability method

At the first level of our analysis, we will focus on two dimensions of XAI methods:

— Local versus global—a local explanation relates to a single prediction (arising from a single input to an ML model), whereas a global explanation seeks to explain the model as a whole [2] thus shedding light on the range of possible predictions.
— Time—we split time for the explanations into three categories: prior—before the prediction is made; contemporaneous—at the same time as the prediction; and post—after the prediction is made.

In §4., we will consider feature-importance and example-based methods. We will also employ the distinction between model-specific and model-agnostic explanation. A model-agnostic explanation can be produced independent of the method used for developing the model, e.g. NNs or SVMs, whereas model-specific explanations depend on the type of ML model used.

## 3. Stakeholders and explanations

### (a) Stakeholders

There are several stakeholder groups who might require an explanation of the ML model and its predictions. Within the scope of this paper, we identify the following classes of stakeholder, each bearing a different relation to the system:

*Prediction-recipients* (e.g. mortgage applicants, offenders in custody, hospital patients). These stakeholders do not use the ML-based system themselves (the prediction is usually mediated by an expert user) but they are directly affected by its predictions.

*End users* (e.g. car drivers, on-line shoppers). These stakeholders are both direct users of the ML-based system and are also directly affected by it. Although the end user will often be a prediction-recipient, we exclude them from that category as they use the system directly. Even so, end users may not always have direct visibility of individual predictions, e.g. for AVs.

*Expert users* (e.g. clinicians, remote pilots). These stakeholders are direct users of the ML-based system but they are not directly affected by its predictions. They are indirectly affected since they may be accountable (both legally and morally) for consequences of enacted predictions.

*Regulatory agencies* (e.g. Financial Services Authority, Vehicle Certification Agency, Medical and Healthcare Products Regulation Agency). These stakeholders are neither direct users of the ML-based system, nor directly affected by it. They do, however, protect the interests of prediction-recipients and end users. The regulatory ecosystem is complex and needs to adapt to ML-based systems [11]. Even so, these bodies are responsible for system approval and deployment; they also oversee the system’s continued (safe) use. Assessors and insurer-funded research centres, e.g. Thatcham, often provide the expert guidance and scrutiny that underpin this regulatory activity.

*Service providers* (e.g. Google, Automated Driving System Entities (ADSE) [12]). These stakeholders are the companies and legal entities who put the system forward for authorization, and vouch for the system when it is deployed. They may be the manufacturer or the software developer, or a joint venture between the two [12]. These stakeholders may be legally liable for the behaviour of the ML-based system once deployed [13].

*Accident and Incident Investigators* (e.g. National Transportation Safety Board (NTSB), Marine Accident Investigation Branch (MAIB), Health and Safety Executive (HSE)). These stakeholders are responsible for analysing accidents or incidents, and for making recommendations for avoiding such events in the future with the same system or similar systems. In some cases, e.g. the HSE, they may also be responsible for initiating legal proceedings;

*Lawyers and the Courts* (e.g. Barristers, Crown Prosecution Service (CPS)). These stakeholders are interested in determining liability for harm caused by an ML-based system. Individual lawyers may seek compensation on behalf of a prediction-recipient or end user.

*Insurers* (e.g. DirectLine, Aviva). These stakeholders cover financial risk on behalf of service providers and users. In practice, they play a useful role to ensure that safety standards are met: they may require evidence that a service provider has met regulatory requirements, and even impose stricter standards of their own.

### (b) Purpose of explanation

Studies of explanations span the sciences, psychology, cognitive science and philosophy [14]. Researchers have noted that the term ‘explanation’ has essentially been re-purposed by the XAI community [15]. What it means in its technical sense touches only on some dimensions of the multi-disciplinary discourse on explanations and their functions.

Explanations provided by XAI methods are descriptive. This speaks to the transparency that the techniques can provide. There are similarities here with scientific modelling. Both deal in approximations that provide descriptions of phenomena or behaviour [15]. XAI methods can also provide causal and logical explanations. They provide some understanding as to how a prediction is generated by the ML model. This speaks to the interpretability that the techniques can provide (hence our use of the term ‘intepretation’ in figure 1). Causality is central to accounts of explanation in philosophy, law, psychology and cognitive science [14]. But philosophical accounts also place an emphasis on normative explanations [16]. These are explanations that offer good reasons for a belief, decision or action; in this way, they can justify a process or an outcome to those affected by it. Explanations provided by XAI methods do not supply explanations in this sense [17]. The methods may, for example, highlight which features in the data have been assigned a larger weight by the model, which determines the effect of a feature on a prediction or the feature’s importance in the model [18]. This assignation of weight is in turn determined by the feature’s success in producing accurate results in the training phase. As such, reasons given for the importance of a certain feature must refer back to human decision-making during the training of the model. This is the wider accountability framework within which XAI methods sit. People, and not (just) systems, are answerable for decisions made in the ML development life cycle.

A great deal of work has been done in the social sciences about people’s expectations from explanations. Some of these, such as the finding that people prefer contrastive explanations accompanied by an underlying causal explanation, can be met or approximated to some degree by XAI methods [14]. Psychological studies indicate an explainer’s values inform explanation selection and evaluation, and these choices in turn can have a significant influence on the recipient’s understanding of an event; the explainer should therefore reflect carefully upon the XAI method used and its communication to the recipient [15]. It is important that explanations are communicated at the appropriate level of abstraction for the stakeholder [19]. The use of visual interfaces can also improve some stakeholders’ epistemic access to an explanation [20]. But equally, interpretations of system behaviour that are presented as more rigorous and complete than they actually are will contribute to unjustified trust [21,22].

Below, we identify some general underlying reasons stakeholders may have for seeking the explanations provided by XAI methods. This characterization is not intended to be understood as homogeneous or exclusive. A single stakeholder, such as an expert user, may have more than one underlying purpose, e.g. they may seek an explanation to determine whether a model complies with regulation as well as an explanation to evaluate confidence in a specific prediction before acting on it. By the same token, individuals from different stakeholder classes may have similar purposes, e.g. to use the information to challenge a particular prediction. Thus our analysis, particularly in §6., should be seen as indicative, not definitive or exhaustive, and it is intended as a starting point on which to build.

Our hypothesis is that understanding these underlying purposes—which we have distilled into the general categories below on the basis of first-hand experience working with developers, industry and regulators—will help to inform understanding of explanation requirements, such as whether a global or local explanation is required, which in turn can inform which XAI methods are most appropriate in a given context. The timing of the explanation will also be relevant to explanation requirements. Our analysis broadly aligns with those in [23,24]. We believe that there would be merit in further empirical study to confirm the nature and importance of the relationships between stakeholder classes and their underlying reasons for seeking explanations of ML-models and predictions.

1. *Clarity*. Greater clarity of the model or its predictions is something all stakeholders, almost by definition, require. It is a prerequisite to meet all the other purposes given below. All dimensions of XAI methods are relevant to answering this need: global explanations prior to deployment; local explanations contemporaneously and local explanations retrospectively. It is also important, however, to temper this requirement with honesty from those providing the XAI methods. They should not be taken to offer clear, or exact, explanations when such clarity is not feasible [21].
2. *Compliance*. Determinations of compliance with law, regulation, or best practice is another underlying purpose to which XAI methods may contribute. Sector-specific regulators will have their own requirements for the approval of ML-based systems. In addition, cross-domain Acts of Parliament apply (e.g. Data Protection Act 2018, UK GDPR, Equality Act 2010). It has been suggested that stakeholders may rely upon XAI methods to fulfil legal duties to provide information about the logic of specific outputs to affected individuals [25]. These will be post-hoc local explanations. In addition, global XAI methods could become part of the toolkit of both regulatory bodies and compliance officers to interrogate and demonstrate the fitness of the system for purpose [26]. And local contemporaneous XAI methods might play a role in ongoing assurance of the model’s performance in context.
3. *Confidence*. Stakeholders will often want to evaluate their confidence in a prediction before proceeding with a decision that has been informed by the prediction. Research suggests that the provision of sufficiently detailed explanations can affect the acceptance of algorithmic decisions by users—primarily expert-users [25]. XAI methods may be used to serve this purpose. Global explanations may inform degrees of confidence in the range of a model’s predictions prior to deployment. Where the ML-based systems are used by human experts to inform decision-making, a local and contemporaneous explanation will be required in order to decide whether to act on a specific prediction in real time.
4. *Consent and Control*. XAI methods may also play a role in enabling stakeholders to better exercise their own human autonomy in relation to an ML-model [24]. Appropriate explanations could enable users to give their informed consent to recommendations by ML-based personal assistants, for example, or, in the case of an AV, to understand a transition demand sufficiently to resume effective hands-on control of the system. This purpose is closely related to confidence, since both ultimately concern acceptance. As with the previous case, the explanations that best serve this purpose will likely be local and contemporaneous.
5. *Challenge*. Stakeholders who seek to challenge or contest a particular prediction may also rely in part upon XAI methods to do so. Examples of members of particular demographic groups being adversely and unfairly affected by ML-based predictions are legion. One particularly egregious example was the COMPAS system, which predicted an individual’s risk of recidivism, and often incorrectly assigned high risk scores to black defendants and low risk scores to white defendants [27]. Other examples include bias in hiring and loan decisions [28]. Demands for fairness often lead to demands for interpretable models [17]. XAI methods may help identify when an error has occurred, or provide evidence to contest a prediction. For such purposes, the requirement will be for a local explanation, after the recommendation has been made.
6. *Continual improvement*. Finally, XAI methods can help developers of ML-based systems, as well as other stakeholders such as accident investigators and regulators, to ensure that the systems are continually improved and updated. The requirement here will be for both global and local explanations.

These different underlying reasons for seeking an explanation are broadly ethical in nature. They relate to the obligations people and organizations owe to one another. They relate to whether people’s reasonable expectations of fairness and respect are met by a model’s predictions. They relate to the exercise of individual human autonomy. They relate to stakeholder assessments of whether a model’s behaviour aligns with normative goals for the system. But necessarily, these purposes will only be served in part by XAI methods. The methods themselves are often approximations, and give partial and selective information. Moreover, XAI methods do not provide normative explanations. They will need to sit within a wider justificatory discourse, in which human organizations and decision-makers provide the reasons for the choices that led to the models being developed as they were throughout the life cycle.

## 4. Explainability methods

Research shows that local XAI methods are far more common than global XAI methods for complex ML models [2]. In this section, we first briefly discuss some relatively simple ML models that are intrinsically interpretable and which can provide both local and global explanations. We then focus on the more complex ML models that tend to be used in critical applications where local XAI methods can provide valuable information. For these more complex models, we look at both feature importance methods, which can be model-agnostic or model-specific, and at example-based methods, which are generally model-agnostic.

### (a) Intrinsically interpretable ML models

Some types of ML model are viewed as being intrinsically interpretable (explainable) due to their simplicity, e.g. linear regression and decision trees [29]. For example, the weights of a linear regression model can be viewed as a crude feature importance score giving global insight into the model if the input features are at a similar scale. The feature importance for a decision tree can be calculated based on the mean decrease of Gini impurity [30] or, as an alternative, using permutation feature importance [31], which calculates importance based on the decrease in the model score when a single feature value is randomly shuffled in the dataset. These explanation methods can provide global insight into the decision tree model, and permutation feature importance has been shown to avoid some flaws of the Gini impurity-based method [32] and is model-agnostic. In addition, these interpretable ML models are often used as a surrogate to approximate other complex ML models giving insight into the more complex ML model [33].

### (b) Explainability methods for complex ML models

There are many different ways to categorize XAI methods, as outlined above. Here, we sub-divide the methods that are useful for complex ML models into feature importance and example-based methods. Feature importance methods can be model-agnostic or model-specific, unlike explanations of intrinsically interpretable models that are normally model-specific. Example-based explanations are normally model-agnostic and are important for explaining the complex ML models used in critical applications. There are many different XAI methods in the literature. We briefly describe some of the more widely used methods here, give an illustrative example in §5. and show how the XAI methods map to stakeholder needs in §6..

### (c) Feature importance methods

Feature importance is by far the most popular method in explainability research [34]. There are two main sub-categories of feature importance methods. One is perturbation-based methods. Another is gradient-based methods. Perturbation-based methods make perturbations to individual inputs either by removing, masking or altering an input feature or set of input features and observing the difference with the original output. This approach can be used in many different applications, e.g. image data, tabular data or text data [35,36]. For example, in an image classification task using CNN, perturbation was implemented by occluding different segments of an input image and visualizing the change in the predicted probability of the classification [37].

*LIME* (Local Interpretable Model-Agnostic Explanations) is a popular pertubation-based method [38]. It generates the explanation by approximating the complex ML model using an interpretable one, e.g. a linear model, learned on perturbations of the single input sample of interest. LIME assumes it is possible to fit an interpretable model around a single input sample that mimics how the complex ML model behaves locally. The simple interpretable model can then be used to explain the predictions of the more complex ML model for this single input sample.

Perturbation methods based on *Shapley values* from cooperative game theory are also very popular [39]. Shapley values are a way to assign the total gain from a cooperative game to its players guaranteeing a unique solution. In using Shapley values to explain a model prediction, the model input features are viewed as the players and the model prediction is the gain resulting from the cooperative game. However, it is difficult to calculate the exact Shapley values in practice as they are exponential in the size of the model input features. Consequently, approximate methods have been proposed, e.g. aggregation-based methods [40], Monte Carlo sampling [41] and approaches for graph-structured data, e.g. language and image data [42].

*SHAP.* (SHapley Additive exPlanations) [43] is another method approximating Shapley values. SHAP incorporates several tools, e.g. KernelSHAP and TreeSHAP [44]. KernelSHAP is a weighted linear regression approximation of the exact Shapley value inspired by LIME and it can be used to provide local explanations for any ML model. TreeSHAP is an efficient estimation approach for tree-based models only, i.e. it is model-specific. The work on SHAP has defined a new class of additive feature importance measures which unifies several existing explainability methods.

Perturbation-based methods allow a direct estimation of feature importance, but they tend to be very slow as they perturb a single input feature or set of features each time, so as the number of input features in the ML model grows, it can take a long time to generate the importance score for all of the features, e.g. for image analysis [45]. Also, as complex ML models are often nonlinear, the explanation is strongly influenced by the set of features that are chosen to be pertubated together. In comparison, gradient-based methods have the potential to be much more efficient.

The basic gradient-based method is just to calculate the gradient of the output with respect to the input. For example, a ‘saliency map’ is produced by calculating the gradient of the output with respect to the input in an image classification task identifying pixels that have a significant influence on the classification [46]. There are several variants of gradient-based methods. Gradient * Input multiplies the gradient (strictly the partial derivative) by the input value to improve the sharpness of feature importance [47]. *Integrated Gradients* is similar to Gradient * Input, in that it computes the gradient of the output with respect to each input feature by integrating over a range from a baseline to the current value of the feature, to produce an average gradient [48]. This method has a number of desirable properties associated with it. *DeepLIFT* (Deep Learning Important FeaTures) [49] has been developed specifically for use with deep NNs. DeepLIFT compares the activation of each neuron to its ‘reference activation’ and assigns an importance score to each input according to the difference. The ‘reference activation’ is obtained through some user-defined reference input to represent an uninformative background value, for example, for image classification this could be a totally black image. DeepLIFT has been shown to be a good approximation to *Integrated Gradients* in most situations [50].

### (d) Example-based methods

Example-based methods use particular input instances to explain complex ML models, thus they normally provide local explanations. This is motivated by the way humans reason, using similar situations to provide explanations [51]. This is common practice, for example, in the law [52,53] where judicial decisions are often based on precedents (known as case law). There is growing interest in using example-based methods to explain complex ML models and some view them as a useful complement to feature-based explanations [54]. We describe three example-based methods.

*Counterfactual explanations* for ML models were introduced by Wachter *et al*. [55]. They use similar situations that give different predictions from the current input instance to the ML model, e.g. achieving a desirable outcome in healthcare. These can be used, for example, to indicate what changes in a patient’s state or treatment are needed to allow them to be discharged from hospital. To be used this way, it is important that the counterfactual explanations minimize the difference between the current input features and the counterfactual examples. The kinds of metrics that should be used to minimize the difference is an ongoing area of research [56,57].

*Adversarial examples* were discovered and discussed by Szegedy *et al.* [58]. They are small, intentional, feature perturbations that cause an ML model to make an incorrect prediction, e.g. to mis-classify an object in image analysis [59] or to fool reading comprehension systems in text classification tasks [60,61]. It is different from counterfactual explanations which are typically used when the ML model is fixed. An adversarial example in autonomous driving might be to add noise to an image of a stop sign so that it would not be recognized by the ML model, although it would seem unchanged to a human. Once such problems are identified, they can be used to improve the robustness of complex ML models. Therefore, adversarial examples are generally used during model training, rather than providing explanations like feature importance methods, but more robust ML models can improve the quality of the feature importance results [62].

*Influential instances* are the inputs from the training dataset that were most influential for the predictions of the ML model, i.e. the ML model parameters are highly influenced by these inputs. One simple way of finding influential examples is to delete inputs from the training dataset, to retrain the model and to assess the impact; while straightforward, this is impractical for large datasets. Often mathematical techniques are used which do not require retraining the model [63]. Like adversarial examples, influential examples are best used during training and contribute more to ML model robustness than providing direct explanations.

## 5. Explainability example

In this section, we present a concrete example, applying a feature importance method (DeepLIFT) to a healthcare application. In Intensive Care Units (ICUs), mechanical ventilation is a complex clinical intervention that consumes a significant proportion of ICU resources [64,65]. It is of critical importance to determine the right time to wean the patient from mechanical support. However, assessment of a patient’s readiness for weaning is a complex clinical task and it is potentially beneficial to employ ML to assist clinicians [66]. The example uses NNs based on the MIMIC-III dataset [67] to predict readiness for weaning in the next hour. The NN models are trained on data for 1839 patient admissions, the NN architecture and hyperparameters are tuned using a validation dataset of 229 patient admissions, and performance is evaluated on 231 patient admissions. For more detail, see [54] which also shows the use of counterfactual explanations.

**Figure 2.**
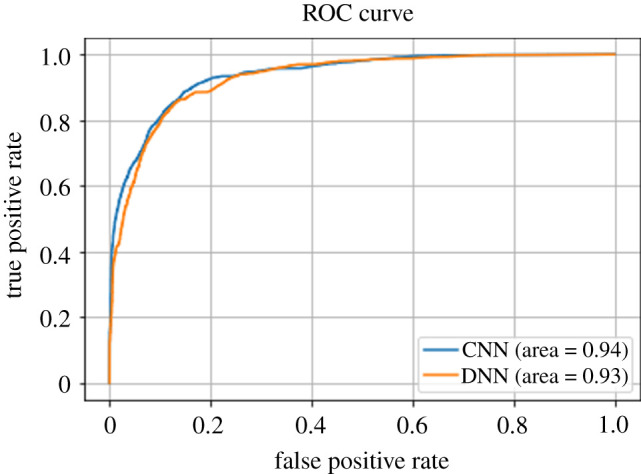
ROC Curves for the example NNs. (Online version in colour.)

**Figure 3.**
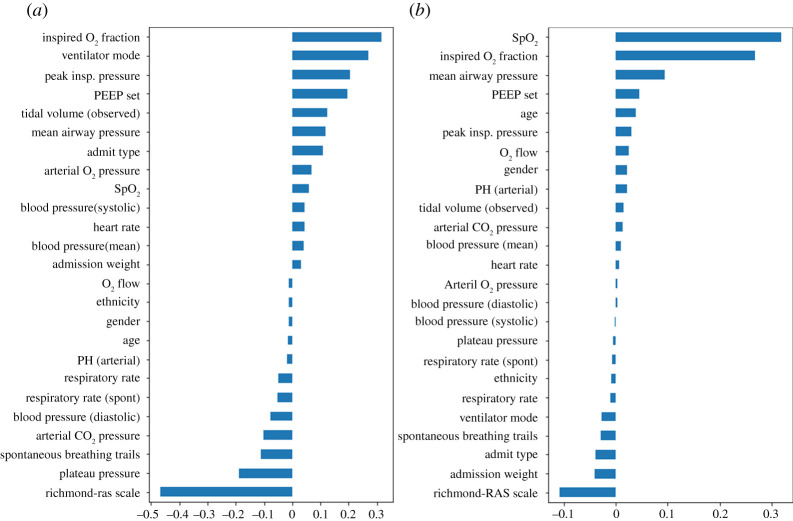
Comparative feature importance. (*a*) CNN feature importance, (*b*) DNN feature importance. (Online version in colour.)

The performance of an ML model is often assessed in terms of accuracy of the prediction and the area under the receiver operating characteristics curve (AUC-ROC). For a ‘random’ model the AUC-ROC would be 0.5 and for a perfect model it would be 1. The example compares both CNN and DNN. Based on performance, both are promising, achieving around 87% accuracy and 0.93–0.94 AUC-ROC (figure 2 and table 1). Based on performance, there seems to be little to choose between them. However, we then used DeepLIFT to determine feature importance for the two NNs, see figure 3 where longer bars mean that the features are of greater importance. Note that the sign indicates positive and negative influences on the outcome and zero means that the feature is of little importance. This shows significant differences between the two models.

**Table 1.** Accuracy for the example NNs.

	accuracy
CNN	86.5%
DNN	87.1%

First, DeepLIFT reveals that the CNN shows ethnicity, gender and age all have an importance near zero, whereas the DNN shows age and gender as having higher importance. Such information should be of critical importance to regulators determining which models to approve. It would be over-simplistic to infer from this information that the CNN is less biased than the DNN, since making models formally ‘blind’ to protected characteristics rarely removes the risk of bias, and may even have the opposite effect, depending on context [68]. But making the feature importance visible to healthcare regulators enables them to ask the right questions about the potential negative impact of proposed ML-based applications on certain demographic groups.

Second, the feature importance for the CNN is more consistent with clinical knowledge. Some features, e.g. the Richardson-RAS scale [69], which show how alert patients are, have high importance in both NNs. However, the CNN places greater importance on a number of patient conditions such as tidal volume (depth of breaths) and on features of the treatment, e.g. the mode of operation of the ventilator, which would typically be considered by a clinician. This use of explainability is an example of decision support for an expert user, but it also has a role in helping ML model developers produce more effective results, and helping regulators determine approval.

This example shows the importance of XAI methods for complex ML models and how the use of performance alone is not enough to assess whether or not a given ML model is an appropriate advisory tool in a safety-critical situation. This also shows how explanations can be visualized, as identified in figure 1, so as to be accessible to stakeholders.

## 6. Integration of stakeholder purposes and XAI methods

The discussion of stakeholders in §3. and the survey of XAI methods in §4. is integrated in table 2. Here, we first explain the table structure then give a more in-depth discussion of three combinations of stakeholder and scenario in which explanations are required, highlighting the ethical considerations for each; we also revisit the example from the previous section showing how it relates to the table.

**Table 2.** Illustrations of explainability requirements for different stakeholders and scenarios.

dimension/example	regulation	investigation	service	service	decision support	decision support
stakeholders	regulator	accident investigator^a^	service provider	end user	expert user	prediction recipient
scenario	system approval	investigate accident or incident	system deployment	service use	decision support	decision support
purpose of explanation	confidence, compliance	clarity, compliance, continuous improvement	confidence, compliance, (continuous improvement)	challenge, consent and control	confidence, consent and control, challenge	challenge
timing of explanations	pre-deployment	post-incident	pre-deployment	same time as decision	same time as decision	same time as decision
data explainability	global	local, global	global	n.a.	local, global	local
model explainability	global (interpretable models, adversarial examples, influential instances)	global (permutation feature importance, counterfactual explanations, TreeSHAP)	global (interpretable models, adversarial examples, influential instances)	n.a.	global (permutation feature importance, interpretable models)	n.a.
prediction explainability	n.a.	local (KernelSHAP, counterfactual explanations)	n.a.	local (KernelSHAP, DeepLIFT, interpretable models)	local (interpretable models, counterfactual explanations)	local (KernelSHAP, DeepLIFT, interpretable models)

^a^Service Provider may investigate service ‘outages’ (incidents) and Lawyers/Courts may also investigate challenges from decision recipients, using similar methods.

Table 2 identifies the stakeholders and the purpose of explanations in a given scenario, e.g. confidence and compliance for a regulator engaged in system approval. Timing for the explanations is identified, and the last three rows correspond to the boxes shaded in gold-brown in figure 1 and identify whether explanations should be local or global, with an *illustration* of candidate XAI methods presented in the last two rows (methods for visualizing data are out of the scope of this paper). The table is intended to be illustrative, not exhaustive. To keep the table compact, investigation covers a number of cases, not just physical accidents. For example, a prediction-recipient might make an immediate challenge to a decision (shown in the right-most column) but the Courts, and a lawyer operating on the recipient’s behalf, might be interested in global as well as local explanations, e.g. to see if an ML model displayed systematic bias.

*Prediction-recipients*, being the directly affected individuals, should always be the core focus of ethics and safety. We also include here directly affected individuals who are excluded from prediction systems. An important ethical consideration for prediction-recipients in domains such as criminal justice and retail banking is that they are not subject to an unfairly discriminatory prediction. There are many examples of ML-based models that have been shown to reinforce bias against individuals on the grounds of race and gender [70], as well as postcode or socio-economic status [71]. While the primary *explananda* here are the human decisions that went into creating the dataset (figure 1), XAI methods will also be required to help to determine whether the ML model reflects or exacerbates bias [72]. As discussed above, feature importance methods can help to determine this. It should be noted that automated predictions about individuals constitute personal data in the case that the individual is identifiable [26]. They therefore fall within the remit of data protection law. This gives rise to requirements for a *post-hoc* local explanation, should the individual seek to *challenge* an automated prediction that has been made about them. This is illustrated in the rightmost column in table 2, showing the relevance of interpretable models to give contemporaneous explanations, but note also the possible need for post-event explanations in support of legal processes.

*Expert-users*, such as radiographers and oncologists, are the individuals for whom most advisory ML-based systems are designed. The objective is for such stakeholders to be able to determine *confidence* in order to make an informed decision about whether to accept and act on the prediction, thus to exercise *consent* and *control*. It is presently unclear whether insufficient scrutiny of an ML-generated prediction would constitute a legal breach of a doctor’s duty of care; however, they may also want to *challenge* or query predictions. But clinicians clearly have a strong moral duty to ensure their patient’s well-being and safety [73]. XAI methods can help them to fulfil that duty. Clinicians make diagnostic decisions by considering and weighing a set of features, data points and clinical markers [74]; see also the example in §5.. The clinician may therefore find value in example-based explanations, such as counterfactual explanations, particularly where their immediate apprehension of the patient, and insights gleaned from additional information not included in the ML model (e.g. biopsy results), indicates a different result to the prediction provided by the system [73].

*Service-providers* have several explanatory requirements, given their need to comply with regulation, ensure the safety of systems, meet the requirements of end users, and to provide explanations to investigators *post-hoc*. This gives rise to a comprehensive range of explanations required, both global explanations prior to deployment for purposes of *confidence*, *compliance* and local explanations *post-hoc* to support *continuous development*. The middle column in table 2 shows the role of XAI methods in the pre-deployment case for the service provider; the column for investigation also covers service providers seeking to understand unintended behaviour and to improve the system.

In addition, the example in §5. is a DSS, and it reflects the requirements for an expert user. In particular, the example shows feature importance methods used to provide model explainability, in support of *Confidence*.

## 7. Discussion

The study of XAI methods is normally conducted from a purely technical perspective. But when, and what type of, explanations are needed, and by which stakeholders, is also often an ethical question. What we have sought to do here is to bring the two dimensions together. The intent is that the information in the form illustrated in table 2 can be used to identify candidate XAI methods, although we acknowledge that the coverage in the table is not exhaustive. Further, this does not produce a ‘unique’ solution and, for example, both LIME and DeepLIFT could be used to provide local explanations for an accident investigation where deep NNs have been used. The development of both ML and XAI methods is proceeding apace so it is unlikely that the choice of XAI methods will be codified in the near future, if indeed that is possible, but it is hoped that an analysis in the form of table 2 will help inform method choice.

Some have argued that only interpretable models should be used for critical decisions [22], and table 2 would seem to support this view. We take a rather broader view that there are trade-offs to be made between performance and explainability. For example, if it is *essential* to understand the model, then the choice of ML methods might be limited, primarily to interpretable models and these have the benefit of providing contemporaneous explanations. But these are less powerful than many other ML methods, and might not perform well in terms of mission objectives. Further, algorithms such as DeepLIFT are fast – the examples shown in figure 3*a*,*b* took around 1–2 min to produce on a modest computer—so these might be useful where a slight delay in producing explanations was acceptable. This suggests that more explicit recognition should be given to XAI methods when choosing the ML methods to use where assurance is a key factor. For example, with AVs, while in theory contemporaneous explanations might be of value, in practice it is doubtful whether or not they would be useful to drivers—and using more powerful ML methods that still permit the use of XAI methods to support incident analysis might be justifiable.

It is generally accepted that verification and validation (V&V) of ML is challenging, and there is no widely agreed ‘best’ way to undertake V&V. Work on building an assurance model for V&V has produced ‘desiderata’ for assurance through the ML life cycle, using a model which inspired the structure of figure 1 [10]. It seems unlikely that assurance processes for ML will reach a level of rigour equivalent to the prevailing standards for ‘conventional software’. Thus, it is possible that explainability will, in time, come to have a greater role in assurance. One of the reasons for believing this is that autonomy, in essence, transfers decision-making from humans to machines—one way to gain confidence that this has been done satisfactorily is to expose the nature of the decision-making process—and this is what explainability seeks to do.

Finally, assurance cases, in the form of structured arguments supported by evidence [75], play a significant role in communicating why it is believed that a system is safe to deploy, particularly in safety-critical industries. The more complex and novel the system and its context are, the more important the role of the assurance case is in informing the risk-based deployment decision. There is an increasing interest in the use of assurance cases to justify the safety of ML-based systems, particularly for automotive [76] and healthcare applications [77]. The notion of explainability, particularly pre-deployment, could form a key part of an ML assurance case used to explain and justify, e.g. to regulators, key decisions about the choice of the ML model and quality and suitability of the data sets. Post-deployment, local XAI methods could help to implement a highly dynamic assurance case [78,79] where the critical predictions made by an ML-based system could be used to update the assumptions about, and confidence in, the system deployed compared with the assessment made pre-deployment.

## 8. Conclusion

ML-based systems are already being used in situations that can have an effect on human well-being, life and liberty. This, combined with the fact that they move decision-making away from humans, makes it an assurance imperative to provide evidence that this transference is appropriate, responsible and safe. Explanations of ML-models and ML-generated predictions can be provided as part of this evidence. But they sit within a wider accountability framework, in which human decision-makers are still required to give the normative reasons or justifications (which XAI methods cannot provide) for the ML-models. Our analysis of stakeholder needs and the contrast with the capabilities of XAI methods gives, we believe, a starting point for understanding how to employ explainability in an assurance role. Assurance of ML-based systems deployed in living environments has an ethical dimension. This is reflected in the underlying ethical nature of the reasons—to inform consent, to challenge an unfair prediction, to assess confidence before implementing a decision that, if wrong, could harm the recipient—for which stakeholders might require visibility of an ML-model or an explanation of one of its predictions. We hope that this paper will help shift the balance of work on XAI methods from a largely technical one to a wider consideration of the role of explainability in assurance and achieving evidence-based acceptance of ML.

## Data accessibility

https://github.com/Yanjiayork/mechanical_ventilator.

## References

[1] Arrieta AB *et al.* 2020 Explainable Artificial Intelligence (XAI): concepts, taxonomies, opportunities and challenges toward responsible AI. Inf. Fusion **58**, 82-115. (10.1016/j.inffus.2019.12.012)

[2] Bhatt U *et al.* 2020 Explainable machine learning in deployment. In *Proc. of the 2020 Conf. on Fairness, Accountability, and Transparency*, pp. 648–657.

[3] McDermid JA. 2010 Safety Critical Software. In *Encyclopedia of Aerospace Engineering* (eds R Blockley, W Shyy). 10.1002/9780470686652.eae506.

[4] Zhang GP. 2000 Neural networks for classification: a survey. IEEE Trans. Syst. Man Cybern. Part C (Appl. Rev.) **30**, 451-462. (10.1109/5326.897072)

[5] Wang G. 2008 A survey on training algorithms for support vector machine classifiers. In *2008 Fourth Int. Conf. on Networked Computing and Advanced Information Management*, vol. 1, *Gyeongju, South Korea, 2–4 September 2008*, pp. 123–128. New York, NY: IEEE.

[6] Arulkumaran K, Deisenroth MP, Brundage M, Bharath AA. 2017 Deep reinforcement learning: a brief survey. IEEE Signal Process Mag. **34**, 26-38. (10.1109/MSP.2017.2743240)

[7] Rawat W, Wang Z. 2017 Deep convolutional neural networks for image classification: a comprehensive review. Neural Comput. **29**, 2352-2449. (10.1162/neco_a_00990) 28599112

[8] Sze V, Chen Y, Yang T, Emer JS. 2017 Efficient processing of deep neural networks: a tutorial and survey. Proc. IEEE **105**, 2295-2329. (10.1109/JPROC.2017.2761740)

[9] Marcus G, Davis E. 2019 Rebooting AI: building artificial intelligence we can trust. London, UK: Vintage.

[10] Ashmore R, Calinescu R, Paterson C. 2019 Assuring the machine learning lifecycle: desiderata, methods, and challenges. http://arxiv.org/abs/190504223.

[11] Centre for Data Ethics and Innovation. Barometer Report. https://assets.publishing.service.gov.uk/government/uploads/system/uploads/attachment_data/file/894170/CDEI_AI_Barometer.pdf. 2020.

[12] Law Commission/The Scottish Law Commission. Automated Vehicles: Consultation Paper 2 on Passenger Services and Public Transport A joint consultation paper. 2019.

[13] Burton S, Habli I, Lawton T, McDermid J, Morgan P, Porter Z. 2020 Mind the gaps: assuring the safety of autonomous systems from an engineering, ethical, and legal perspective. Artif. Intell. **279**, 103201. (10.1016/j.artint.2019.103201)

[14] Miller T. 2019 Explanation in artificial intelligence: insights from the social sciences. Artif. Intell. **267**, 1-38. (10.1016/j.artint.2018.07.007)

[15] Mittelstadt B, Russell C, Wachter S. 2019 Explaining explanations in AI. In *Proc. of the Conf. on fairness, accountability, and transparency, Atlanta, GA, 29–31 January 2019*, pp. 279–288. New York, NY: ACM.

[16] Raz J. 2011 From normativity to responsibility. Oxford, UK: Oxford University Press.

[17] Lipton ZC. 2018 The mythos of model interpretability: in machine learning, the concept of interpretability is both important and slippery. Queue **16**, 31-57. (10.1145/3236386.3241340)

[18] Biran O, McKeown K. 2014 Justification narratives for individual classifications. In *Proc. of the AutoML workshop at ICML*, vol. 2014, pp. 1–7.

[19] Ward FR, Habli I. 2020 An assurance case pattern for the interpretability of machine learning in safety-critical systems. In *Int. Conf. on Computer Safety, Reliability, and Security, Lisbon, Portugal, 15–18 September 2020*, pp. 395–407. Berlin, Germany: Springer.

[20] Tsamados A, Aggarwal N, Cowls J, Morley J, Roberts H, Taddeo M, Floridi L. 2021 The ethics of algorithms: key problems and solutions. AI & SOCIETY 1-16.

[21] Brundage M *et al.* 2020 Toward trustworthy AI development: mechanisms for supporting verifiable claims. http://arxiv.org/abs/200407213.

[22] Rudin C. 2019 Stop explaining black box machine learning models for high stakes decisions and use interpretable models instead. Nat. Mach. Intell. **1**, 206-215. (10.1038/s42256-019-0048-x) PMC9122117 35603010

[23] Weller A. 2019 Transparency: motivations and challenges. In *Explainable AI: interpreting, explaining and visualizing deep learning* (eds W Samek, G Montavon, A Vedaldi, LK Hansen, K-R Müller), pp. 23–40. Berlin, Germany: Springer.

[24] Langer M, Oster D, Speith T, Hermanns H, Kästner L, Schmidt E, Sesing A, Baum K. 2021 What do we want from Explainable Artificial Intelligence (XAI)? A stakeholder perspective on XAI and a conceptual model guiding interdisciplinary XAI research. Artif. Intel. **296**, 103473. (10.1016/j.artint.2021.103473)

[25] Binns R, Van Kleek M, Veale M, Lyngs U, Zhao J, Shadbolt N. 2018 ‘It’s Reducing a Human Being to a Percentage’ perceptions of justice in algorithmic decisions. In *Proc. of the 2018 Chi Conf. on human factors in computing systems, Montreal, Canada, 21–26 April 2018*, pp. 1–14. New York, NY: ACM.

[26] Information Commissioners Office & Alan Turing Institute. 2020 Explaining Decisions made with AI. See .

[27] Freeman K. 2016 Algorithmic injustice: how the Wisconsin Supreme Court failed to protect due process rights in State v. Loomis. North Carolina. J. Law Technol. **18**, 75.

[28] Barocas S, Selbst AD. 2016 Big data’s disparate impact. Calif. L. Rev. **104**, 671.

[29] Hastie T, Tibshirani R, Friedman J. 2009 The elements of statistical learning. Berlin, Germany: Springer.

[30] Louppe G. 2014 Understanding random forests: from theory to practice. http://arxiv.org/abs/14077502.

[31] Breiman L. 2001 Random forests. Mach. Learn. **45**, 5-32. (10.1023/A:1010933404324)

[32] Parr T, Turgutlu K, Csiszar C, Howard J. 2018 Beware default random forest importances. March **26**, 2018.

[33] Molnar C. 2020 Interpretable machine learning. See Lulu.com.

[34] Gilpin LH, Bau D, Yuan BZ, Bajwa A, Specter M, Kagal L. 2018 Explaining explanations: an overview of interpretability of machine learning. In *2018 IEEE 5th Int. Conf. on data science and advanced analytics (DSAA), Turin, Italy, 1–3 October 2018*, pp. 80–89. New York, NY: IEEE.

[35] Montano J, Palmer A. 2003 Numeric sensitivity analysis applied to feedforward neural networks. Neural Comput. Appl. **12**, 119-125. (10.1007/s00521-003-0377-9)

[36] Liang B, Li H, Su M, Bian P, Li X, Shi W. 2017 Deep text classification can be fooled. http://arxiv.org/abs/170408006.

[37] Zeiler MD, Fergus R. 2014 Visualizing and understanding convolutional networks. In *European Conf. on computer vision, Zürich, Switzerland, 6–12 September 2014*, pp. 818–833. Berlin, Germany: Springer.

[38] Ribeiro MT, Singh S, Guestrin C. 2016 ‘Why should I trust you?’ Explaining the predictions of any classifier. In *Proc. of the 22nd ACM SIGKDD Int. Conf. on knowledge discovery and data mining, San Francisco, CA, 13–17 August 2016*, pp. 1135–1144. New York, NY: ACM.

[39] Shapley LS. 1953 A value for n-person games. Contrib. Theory Games **2**, 307-317.

[40] Bhatt U, Ravikumar P, Moura JM. 2019 Towards aggregating weighted feature attributions. http://arxiv.org/abs/190110040.

[41] Štrumbelj E, Kononenko I. 2014 Explaining prediction models and individual predictions with feature contributions. Knowl. Inf. Syst. **41**, 647-665. (10.1007/s10115-013-0679-x)

[42] Chen J, Song L, Wainwright MJ, Jordan MI. 2018 L-shapley and c-shapley: efficient model interpretation for structured data. http://arxiv.org/abs/180802610.

[43] Lundberg SM, Lee SI. 2017 A unified approach to interpreting model predictions. In *Advances in neural information processing systems* (eds U von Luxburg, I Guyon, S Bengio, H Wallach, R Fergus), pp. 4765–4774.

[44] Lundberg SM *et al.* 2020 From local explanations to global understanding with explainable AI for trees. Nat. Mach. Intell. **2**, 2522-5839. (10.1038/s42256-019-0138-9) PMC7326367 32607472

[45] Zintgraf LM, Cohen TS, Adel T, Welling M. 2017 Visualizing deep neural network decisions: prediction difference analysis. http://arxiv.org/abs/170204595.

[46] Simonyan K, Vedaldi A, Zisserman A. 2013 Deep inside convolutional networks: visualising image classification models and saliency maps. http://arxiv.org/abs/13126034.

[47] Shrikumar A, Greenside P, Shcherbina A, Kundaje A. 2016 Not just a black box: learning important features through propagating activation differences. http://arxiv.org/abs/160501713.

[48] Sundararajan M, Taly A, Yan Q. 2017 Axiomatic attribution for deep networks. http://arxiv.org/abs/170301365.

[49] Shrikumar A, Greenside P, Kundaje A. 2017 Learning important features through propagating activation differences. http://arxiv.org/abs/170402685.

[50] Ancona M, Ceolini E, Öztireli C, Gross M. 2017 Towards better understanding of gradient-based attribution methods for deep neural networks. http://arxiv.org/abs/171106104.

[51] Aamodt A, Plaza E. 1994 Case-based reasoning: foundational issues, methodological variations, and system approaches. AI Commun. **7**, 39-59. (10.3233/AIC-1994-7104)

[52] Kolodner JL. 1992 An introduction to case-based reasoning. Artif. Intell. Rev. **6**, 3-34. (10.1007/BF00155578)

[53] Richter MM, Weber RO. 2016 Case-based reasoning. Berlin, Germany: Springer.

[54] Jia Y, Kaul C, Lawton T, Murray-Smith R, Habli I. 2020 Prediction of weaning from mechanical ventilation using convolutional neural networks. Artif. Intell. Med. **117**, 102087. 10.1016/j.artmed.2021.102087 34127233

[55] Wachter S, Mittelstadt B, Russell C. 2017 Counterfactual explanations without opening the black box: automated decisions and the GDPR. Harv. JL & Tech. **31**, 841. (10.2139/ssrn.3063289)

[56] Mothilal RK, Sharma A, Tan C. 2020 Explaining machine learning classifiers through diverse counterfactual explanations. In *Proc. of the 2020 Conf. on Fairness, Accountability, and Transparency, Barcelona, Spain, 27–30 January 2020*, pp. 607–617.

[57] Sharma S, Henderson J, Ghosh J. 2019 Certifai: Counterfactual explanations for robustness, transparency, interpretability, and fairness of artificial intelligence models. http://arxiv.org/abs/190507857.

[58] Szegedy C, Zaremba W, Sutskever I, Bruna J, Erhan D, Goodfellow I, Fergus R. 2013 Intriguing properties of neural networks. http://arxiv.org/abs/13126199.

[59] Xie C, Tan M, Gong B, Wang J, Yuille AL, Le QV. 2020 Adversarial examples improve image recognition. In *Proc. of the IEEE/CVF Conf. on Computer Vision and Pattern Recognition, Seattle, WA, 13–19 June 2020*, pp. 819–828. New York, NY: IEEE.

[60] Jia R, Liang P. 2017 Adversarial examples for evaluating reading comprehension systems. http://arxiv.org/abs/170707328.

[61] Sato M, Suzuki J, Shindo H, Matsumoto Y. 2018 Interpretable adversarial perturbation in input embedding space for text. http://arxiv.org/abs/180502917.

[62] Etmann C, Lunz S, Maass P, Schönlieb CB. 2019 On the connection between adversarial robustness and saliency map interpretability. http://arxiv.org/abs/190504172.

[63] Koh PW, Liang P. 2017 Understanding black-box predictions via influence functions. http://arxiv.org/abs/170304730.

[64] Wunsch H, Wagner J, Herlim M, Chong D, Kramer A, Halpern SD. 2013 ICU occupancy and mechanical ventilator use in the United States. Crit. Care Med. **41**, 2712-2719. (10.1097/CCM.0b013e318298a139) 23963122 PMC3840149

[65] Ambrosino N, Gabbrielli L. 2010 The difficult-to-wean patient. Expert Rev. Respiratory Med. **4**, 685-692. (10.1586/ers.10.58) 20923345

[66] Kuo HJ, Chiu HW, Lee CN, Chen TT, Chang CC, Bien MY. 2015 Improvement in the prediction of ventilator weaning outcomes by an artificial neural network in a medical ICU. Respir. Care **60**, 1560-1569. (10.4187/respcare.03648) 26329358

[67] Johnson AE *et al.* 2016 MIMIC-III, a freely accessible critical care database. Sci. Data **3**, 1-9. (10.1038/sdata.2016.35) PMC4878278 27219127

[68] Kroll JA, Barocas S, Felten EW, Reidenberg JR, Robinson DG, Yu H. 2016 Accountable algorithms. U. Pa. L. Rev. **165**, 633.

[69] Sessler CN, Gosnell MS, Grap MJ, Brophy GM, O’Neal PV, Keane KA, Tesoro EP, Elswick RK. 2002 The Richmond Agitation–Sedation Scale: validity and reliability in adult intensive care unit patients. Am. J. Respir. Crit. Care Med. **166**, 1338-1344. (10.1164/rccm.2107138) 12421743

[70] Mehrabi N, Morstatter F, Saxena N, Lerman K, Galstyan A. 2019 A survey on bias and fairness in machine learning. http://arxiv.org/abs/190809635.

[71] Eubanks VA. 2018 *Automating inequality: how high-tech tools profile, police, and punish the poor*. New York, NY: St. Martin’s Press.

[72] Centre for Data Ethics and Innovation. Review into Bias in Algorithmic Decision-Making; 2020. See https://assets.publishing.service.gov.uk/government/uploads/system/uploads/attachment_data/file/939109/CDEI_review_into_bias_in_algorithmic_decision-making.pdf.

[73] Habli I, Lawton T, Porter Z. 2020 Artificial intelligence in health care: accountability and safety. Bull. World Health Organ. **98**, 251. (10.2471/BLT.19.237487) 32284648 PMC7133468

[74] Sullivan E. 2019 Understanding from machine learning models. Br. J. Philos. Sci. (10.1093/bjps/axz035)

[75] Kelly TP. Arguing safety: a systematic approach to managing safety cases. PhD thesis, University of York.

[76] Burton S, Gauerhof L, Heinzemann C. 2017 Making the case for safety of machine learning in highly automated driving. In *Int. Conf. on Computer Safety, Reliability, and Security, Trento, Italy, 12–15 September 2017*, pp. 5–16. Berlin, Germany: Springer.

[77] Picardi C, Hawkins R, Paterson C, Habli I. 2019 A pattern for arguing the assurance of machine learning in medical diagnosis systems. In *Int. Conf. on Computer Safety, Reliability, and Security*, pp. 165–179. Berlin, Germany: Springer.

[78] Denney E, Pai G, Habli I. 2015 Dynamic safety cases for through-life safety assurance. In *2015 IEEE/ACM 37th IEEE Int. Conf. on Software Engineering, Florence, Italy, 16–24 May 2015*, vol. 2, pp. 587–590. New York, NY: IEEE.

[79] Asaadi E, Denney E, Menzies J, Pai GJ, Petroff D. 2020 Dynamic assurance cases: a pathway to trusted autonomy. Computer **53**, 35-46. (10.1109/MC.2020.3022030)

